# Robot-assisted lumbar fixation in single lateral position: OLIF L4–5, ALIF L5–S1, and L4–S1 percutaneous pedicle screws

**DOI:** 10.3171/2025.4.FOCVID257

**Published:** 2025-07-01

**Authors:** Mario Taravilla-Loma, Javier Giner García, Víctor Rodríguez-Domínguez, Pablo García-Feijoo, María Luisa Gandía-González, Alberto Isla Guerrero

**Affiliations:** Department of Neurosurgery, Hospital Universitario La Paz, Madrid, Spain

**Keywords:** robotic spine surgery, single lateral position, interbody fusion, minimally invasive spine surgery

## Abstract

The integration of robot-assisted spine surgery (RASS) and single lateral position surgery (SLPS) has revolutionized spinal fusion by enhancing efficiency, reducing surgical time, and improving patient outcomes. This video demonstrates a step-by-step procedural workflow for robot-assisted OLIF L4–L5, ALIF L5–S1, and percutaneous pedicle screw fixation (L4–S1) in a single lateral decubitus position using the ExcelsiusGPS system. The workflow highlights preoperative CT-based planning, intraoperative image fusion, real-time navigation, and robot-guided implant placement. SLPS eliminates the need for patient repositioning, reduces anesthesia duration, and enhances operating room efficiency, showcasing the role of robotics and innovative positioning strategies in modern spine surgery.

The video can be found here: https://stream.cadmore.media/r10.3171/2025.4.FOCVID257

## Transcript

### 0:25 Robot-Assisted Spine Surgery (RASS) and Single Lateral Position Surgery (SLPS).

Robot-assisted spine surgery has emerged as one of the most significant technological advances in spinal procedures.^1^ Among the different robotic systems available, this video presents our experience with the ExcelsiusGPS robotic platform, having performed over 150 spine surgeries at our institution.^2^ The ExcelsiusGPS system provides both preoperative and intraoperative workflows. However, this discussion will focus on the preoperative workflow. The preoperative workflow requires a volumetric preoperative spinal CT scan with a slice thickness of < 1 mm. Using this CT scan, we generate a preoperative surgical plan in the workstation, determining the trajectory and size of the pedicle screws.^3^ At the beginning of surgery, after positioning the patient, we acquire lateral and anteroposterior x-rays, which are merged with the preoperative CT scan to facilitate accurate image fusion.^4^ The pedicle screws and interbody cages are subsequently placed according to the preplanned trajectories, ensuring optimal alignment and fixation. The single lateral position surgery (SLPS) for interbody fusion and percutaneous pedicle screw fixation offers multiple advantages compared to conventional repositioning-based surgery. For surgeons, it streamlines the surgical workflow, enhances procedural efficiency, and minimizes intraoperative disruptions. For patients, it results in reduced anesthesia time, less blood loss, lower fluoroscopic radiation exposure, shorter operative time, decreased postoperative morbidity, and faster recovery.^3,4^ For hospitals, SLPS enhances operating room efficiency and reduces patient length of stay.^5^ However, some limitations exist, including the requirement for robotic or navigation systems, an uncomfortable positioning setup, and the need for a learning curve to adapt to the new orientation.^6^

### 2:23 Clinical Case: Background and Neurological Exam. Imaging Findings and Electromyography (EMG).

A 73-year-old female with a history of obesity (BMI 38) presented with chronic low back pain and right-sided sciatica, progressively worsening over the past year. Neurological examination was unremarkable. Preoperative MRI revealed Meyerding grade I degenerative spondylolisthesis at L4–5, accompanied by Pfirrmann’s grade IV disc degeneration and moderate-to-severe left foraminal and recess stenosis. At L5–S1, Pfirrmann’s grade V disc degeneration with severe right recess and foraminal stenosis. Electromyography (EMG) showed fibrillation potentials and reduced recruitment in the L4–5 roots, suggestive of neurological involvement at that level (Supplementary Table 1; Supplementary Fig. 1).

### 3:19 Surgical Planning.

Using the preoperative workflow, we precisely mapped the screw trajectories and determined the optimal interbody cage placement on the robotic workstation. This allows for optimal implant positioning and surgical efficiency.^7^

### 3:37 Surgery.

The patient was positioned in a right lateral decubitus position.^8^ The upper leg was extended, while the lower leg was slightly flexed. The surgery was performed in two stages: first, percutaneous pedicle screw placement (L4–S1) with ExcelsiusGPS. Subsequently, OLIF L4–5 and ALIF L5–S1. The OLIF L4–5 skin incision is made 2 or 3 cm anterior to the anterior disc margin of L4–5, while the ALIF L5–S1 incision is placed 3 or 4 cm anterior to the anterior superior iliac spine (ASIS).^6^

### 4:23 Stage 1: Robotic Landmarks Placement and Vertebral Level Identification.

Robotic landmarks were placed on the iliac crest, including the dynamic reference base (DRB), which maintains real-time patient tracking to adjust for movement, and the surveillance tracker, which detects involuntary movements and updates navigation accordingly.^2,3^ In the preoperative workflow, anteroposterior and lateral radiographs are initially taken to identify each vertebra to be instrumented. Its objective is to carry out an adequate registration and alignment of the patient within the navigation system.^4^

### 5:03 Stage 1: Fusion of X-Ray and Preoperative CT Images With Merge Verification.

The robotic system processed the preoperative CT scan to extract vertebral landmarks, facilitating precise alignment with intraoperative x-rays for accurate image fusion. Subsequently, the merge must be checked.^2^

### 5:21 Stage 1: Transpedicular Screw Placement.

The robotic arm was aligned to ensure precise screw insertion according to the preplanned trajectory.^6^ Entry points were marked on the skin, followed by a small incision to expose the muscle fascia. Pedicle drilling was performed using the robot-assisted drill, ensuring controlled cortical penetration.^7^ Tap threading was applied to prepare the screw trajectory, optimizing bone purchase and stability. Finally, transpedicular screws were inserted and secured in their designated positions.^8^

### 6:25 Stage 2: OLIF L4–5.

In the second stage of the procedure, OLIF L4–5 and ALIF L5–S1 were performed. To perform the OLIF L4–5, the skin incision is made 2 or 3 cm anterior to the leading edge of the L4–5 disc space. After exposing and sectioning the muscle fascia, blunt dissection of the abdominal wall musculature was performed, sequentially passing through the external oblique, internal oblique, and transversus abdominis muscle, until reaching the retroperitoneal space.^4^ The retroperitoneal space was accessed, identifying the genitofemoral nerve first, followed by the psoas muscle.^5^ The cage trajectory is renavigated, confirming its position in the prepsoas L4–5 space.^6^ Next, the L4–5 annulus is incised, followed by discectomy.^8^ The L4–5 disc space is prepared, ensuring contralateral release to restore intervertebral height.^3^ The interbody cage is inserted under navigation guidance, following the planned trajectory.^5^ An orthogonal adjustment maneuver is performed to optimize cage positioning, ensuring precise alignment under intraoperative navigation guidance. The final interbody cage is implanted and secured. Retractors are removed, and muscular layers are visualized, confirming proper anatomical restoration.

### 8:04 Stage 2: ALIF L5–S1.

Following the OLIF L4–5, we proceed with the ALIF L5–S1. Skin incision is placed 3 or 4 cm anterior to the anterior superior iliac spine (ASIS). A layered dissection is performed, progressively exposing the muscular fascia, which is sectioned. A blunt dissection is performed through the abdominal wall musculature. The retroperitoneal space is accessed. Within the retroperitoneal corridor, anatomical structures are carefully identified: the left ureter is visualized and mobilized. Further dissection reveals the left common iliac artery and vein. The sacral promontory and L5–S1 disc space are identified. The L5–S1 annulus is incised, followed by L5–S1 discectomy and disc space preparation. A trial interbody cage is inserted to assess the fit and positioning. The interbody cage was packed with demineralized bone matrix putty prior to placement. Subsequently, the final cage was implanted.

### 9:16 Titanium Rod Placement.

Titanium rods are securely placed to stabilize the pedicle screws.

### 9:21 Four-Hand Closure and Postoperative Images.

A four-hand closure technique is performed, allowing both surgeons to close the surgical site simultaneously, increasing efficiency.^3^ Postoperative images are obtained to confirm correct implant positioning and alignment.

### 9:40 Follow-Up.

At the 3-month postoperative follow-up, the patient exhibits proper wound healing with no signs of complications. There is significant clinical improvement, with a Visual Analog Scale (VAS) pain score reduction from 8/10 to 2/10. Significant functional recovery is noted, with the Oswestry Disability Index (ODI) improving from 60% to 15%, demonstrating a positive surgical outcome and effective rehabilitation (Supplementary Fig. 2).

## Acknowledgments

An artificial intelligence–based voice modification tool (CapCut) was used to enhance the audio narration of the surgical video. No AI-generated content was used in the creation of the manuscript text, figures, or scientific content. All medical and surgical information was developed and verified by the authors.

## Disclosures

The authors report no conflict of interest concerning the materials or methods used in this study or the findings specified in this publication.

## Author Contributions

Primary surgeon: Giner García. Assistant surgeon: Taravilla-Loma. Editing and drafting the video and abstract: Taravilla-Loma, Rodríguez-Domínguez, García-Feijoo, Gandía-González. Critically revising the work: Taravilla-Loma, Rodríguez-Domínguez, García-Feijoo, Gandía-González. Reviewed submitted version of the work: Taravilla-Loma, García-Feijoo, Gandía-González. Approved the final version of the work on behalf of all authors: Taravilla-Loma. Supervision: García-Feijoo, Gandía-González, Isla Guerrero.

## Supplemental Information

Supplementary Table and Figures

### Online-Only Content

Supplemental material is available online.

*Supplementary Table and Figures.* https://thejns.org/doi/suppl/10.3171/2025.4.FOCVID257.

### Patient Informed Consent

The necessary patient informed consent was obtained in this study.
