# Supplementary material for: Robot-assisted lumbar fixation in single lateral position: OLIF L4–5, ALIF L5–S1, and L4–S1 percutaneous pedicle screws
Source: Neurosurg Focus Video. 2025 Jul 1;13(1):V4. doi: 10.3171/2025.4.FOCVID257 (PMC12308739; doi:10.3171/2025.4.FOCVID257)
Supplement: Supplementary Table and Figures [file SupplementaryTableandFigures_FOCVID25-7.pdf]

NEUROSURGICAL FOCUS VIDEO



ONLINE ONLY

# Supplemental material

## Robot-assisted lumbar fixation in single lateral position: OLIF L4–5, ALIF L5–S1, and L4–S1 percutaneous pedicle screws

Taravilla-Loma et al.

https://thejns.org/doi/abs/10.3171/2025.4.FOCVID257

**DISCLAIMER** The *Journal of Neurosurgery* acknowledges that the following section is published verbatim as submitted by the authors and did not go through either the *Journal's* peer-review or editing process.

© 2025 The authors, CC BY 4.0 (http://creativecommons.org/licenses/by/4.0/)

| MUSCLE | NERVE | ROOTS | SPONTANEOUS ACTIVITY | FIBRILLATIONS | PSW | FASCICULATIONS | HIGH FREQUENCY (H.F.) | PUMS AMPLITUDE | PUMS DURATION | POLYPHASIC PUMS | RECRUITMENT | PATTERN |
|---|---|---|---|---|---|---|---|---|---|---|---|---|
| R. Vastus medialis | Femoral | L2-L4 | N | N | N | No | No | N | N | N | Interfering | Normal |
| R. Tibialis anterior | Deep peroneal | L4-L5 | N | 2+ | 2+ | No | No | 1+ | 1+ | N | Mixed-Poor | Abnormal |
| R. Tibialis posterior | Tibial | L4-L5 | N | 1+ | 1+ | No | No | 1+ | 1+ | N | Mixed-Poor | Abnormal |
| R. Gastrocnemius (medial head) | Tibial | S1-S2 | N | N | N | No | No | N | N | N | Interfering | Normal |

**Supplementary Table 1. Electromyography (EMG) results showing nerve root impairment at L4-L5, with fibrillation and reduced recruitment patterns.**

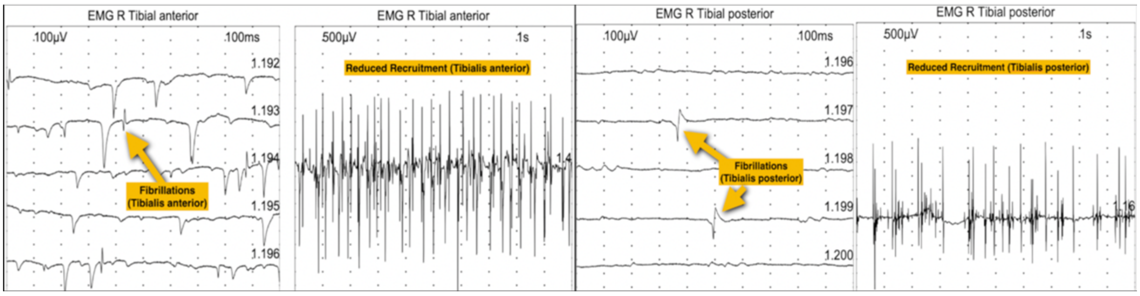


**Supplementary Figure 1. Electromyography (EMG) graphs illustrating fibrillation and reduced motor unit recruitment at affected spinal levels.**

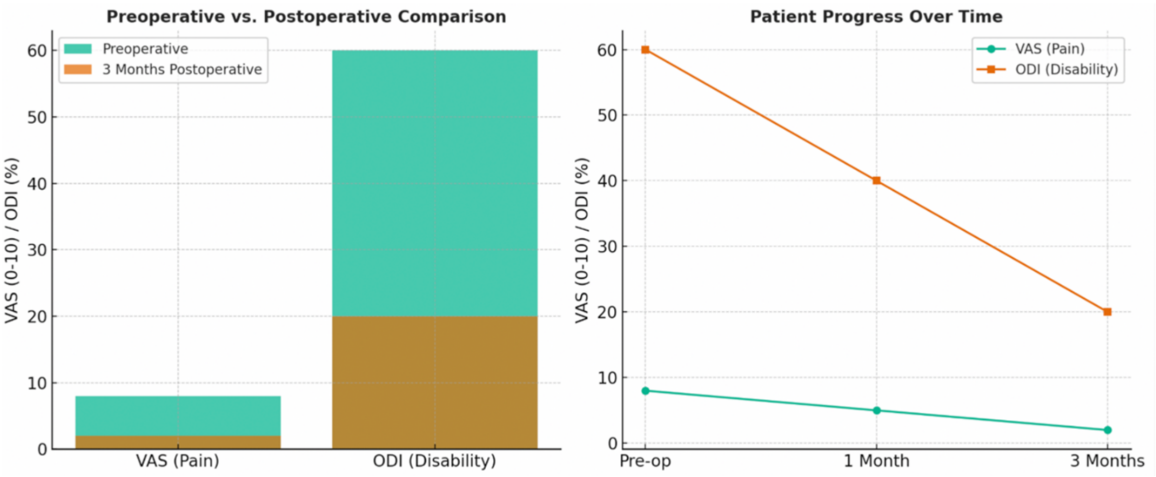


**Supplementary Figure 2. Postoperative VAS and ODI scores, demonstrating pain reduction and functional improvement at 3 months.**
